# Whole-genome sequencing and functional annotation of pathogenic *Paraconiothyrium brasiliense* causing human cellulitis

**DOI:** 10.1186/s40246-023-00512-5

**Published:** 2023-07-17

**Authors:** Haibing Liu, Yue Zhang, Jianguo Chen

**Affiliations:** Department of Clinical Laboratory, The Affiliated People’s Hospital of Jiangsu University, Zhenjiang, Jiangsu China

**Keywords:** *Paraconiothyrium brasiliense*, Whole-genome sequencing, Functional annotation, Human pathogenic fungus

## Abstract

**Background:**

A pathogenic filamentous fungus causing eyelid cellulitis was isolated from the secretion from a patient's left eyelid, and a phylogenetic analysis based on the rDNA internal transcribed spacer region (ITS) and single-copy gene families identified the isolated strain as *Paraconiothyrium brasiliense*. The genus *Paraconiothyrium* contains the major plant pathogenic fungi, and in our study, *P. brasiliense* was identified for the first time as causing human infection. To comprehensively analyze the pathogenicity, and proteomics of the isolated strain from a genetic perspective, whole-genome sequencing was performed with the Illumina NovaSeq and Oxford Nanopore Technologies platforms, and a bioinformatics analysis was performed with BLAST against genome sequences in various publicly available databases.

**Results:**

The genome of *P. brasiliense* GGX 413 is 39.49 Mb in length, with a 51.2% GC content, and encodes 13,057 protein-coding genes and 181 noncoding RNAs. Functional annotation showed that 592 genes encode virulence factors that are involved in human disease, including 61 lethal virulence factors and 30 hypervirulence factors. Fifty-four of these 592 virulence genes are related to carbohydrate-active enzymes, including 46 genes encoding secretory CAZymes, and 119 associated with peptidases, including 70 genes encoding secretory peptidases, and 27 are involved in secondary metabolite synthesis, including four that are associated with terpenoid metabolism.

**Conclusions:**

This study establishes the genomic resources of *P. brasiliense* and provides a theoretical basis for future studies of the pathogenic mechanism of its infection of humans, the treatment of the diseases caused, and related research.

**Supplementary Information:**

The online version contains supplementary material available at 10.1186/s40246-023-00512-5.

## Background

The genus *Paraconiothyrium* includes 27 species and belongs to the family Sphaeropsidaceae in the order Sphaeropsidales and class Coelomycetes [1]. The species of the genus *Paraconiothyrium* have a wide range of lifestyles, including in soil, marine environments [2], and the digestive tracts of insects [3]. The genus *Paraconiothyrium* is often described as containing pathogens of plants and opportunistic pathogens of humans. According to reports, the branch canker of hawthorn tree is caused by *P. sporulosum* [4], and *P. variabile* causes leaf spot when it infects the leaves of *Phoenix theophrasti* [5]. Moreover, a man with end-stage renal disease and a cadaveric renal transplant developed cutaneous phaeohyphomycosis caused by *P. cyclothyrioides* [6], and a 71-year-old man who had undergone kidney transplantation developed a cellulitic lesion caused by *P. cyclothyrioides* [7].

*Paraconiothyrium brasiliense* was first isolated from the fruit of *Coffea arabica* by M. Taniwaki in Brazil and was first described by Verkley et al. in 2004 [8]. Like other species of *Paraconiothyrium*, *P. brasiliense* expresses various biological activities and has potential applications in many fields. For instance, *P. brasiliense* isolated from Himalayan yew produces paclitaxel, with antitumor activity [9]. Arredondo-Santoyo et al. [10] showed that *P. brasiliense* expresses an activity antagonistic to *Colletotrichum* spp. and *Phytophthora* spp. Four tricyclic sesquiterpenoids produced by a *P. brasiliense* strain isolated from *Acer truncatum* showed effective activity against *Human immunodeficiency virus 1* (HIV-1) [11]. Afshan et al. were the first to report that *P. brasiliense* causes leaf spot of *Sarcococca saligna* in Pakistan, but there have been no reports as yet of *P. brasiliense* causing disease in humans.

In this study, we isolated a pathogenic *P. brasiliense* strain causing cellulitis in a patient with a tree branch injury to the left eyelid. To provide a theoretical basis from which to study the pathogenesis, proteomics, and transcriptomics of this pathogenic *P. brasiliense* from the perspective of genetics, the whole genome was sequenced with next-generation sequencing and third-generation sequencing technologies. We performed a bioinformatics analysis based on the whole-genome sequence, including the functional annotation of protein-coding genes, taxonomic identification, the prediction of pathogenic factors, and the analysis of repetitive sequences and noncoding RNA (ncRNA).

## Results

### Morphology and identification of isolated strain

**Fig. 1:**
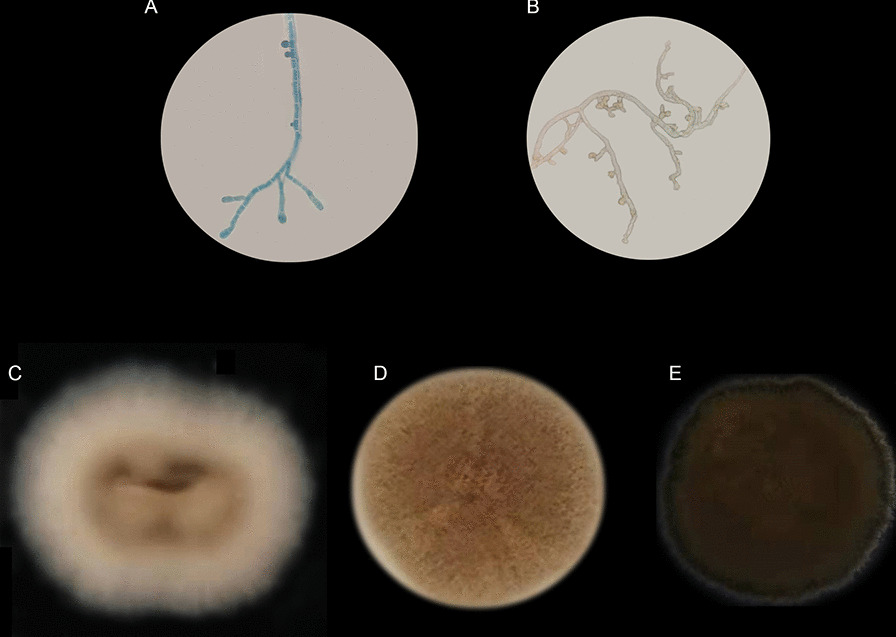
Morphology of filamentous fungus. **A** Mycelium stained with lactophenol cotton blue. **B** Morphology of the unstained mycelium. **C** Colony morphology after 4 days in culture on SDA medium. **D** Colony morphology after 8 days in culture on SDA medium. **E** Colony morphology after 12 days in culture on SDA medium

The patient was diagnosed with cellulitis of the left eyelid caused by a filamentous fungus, the mycelium of which was septate (Fig. 1A and B). The fungal colony on SDA medium was round, with a neat edge, low convexity, and an even to slightly ruffled colony surface covered by felty floccose. The colony was white at the edge, darkening from the edge to the center, and the aerial hyphae changed from white to dark brown. The diameter of the colony was 16 mm after 4 days (Fig. 1C), 42 mm after 8 days (Fig. 1D), and 60 mm after 12 days (Fig. 1E). The filamentous fungus was designated GGX 413 based on information about the infected patient.

**Fig. 2:**
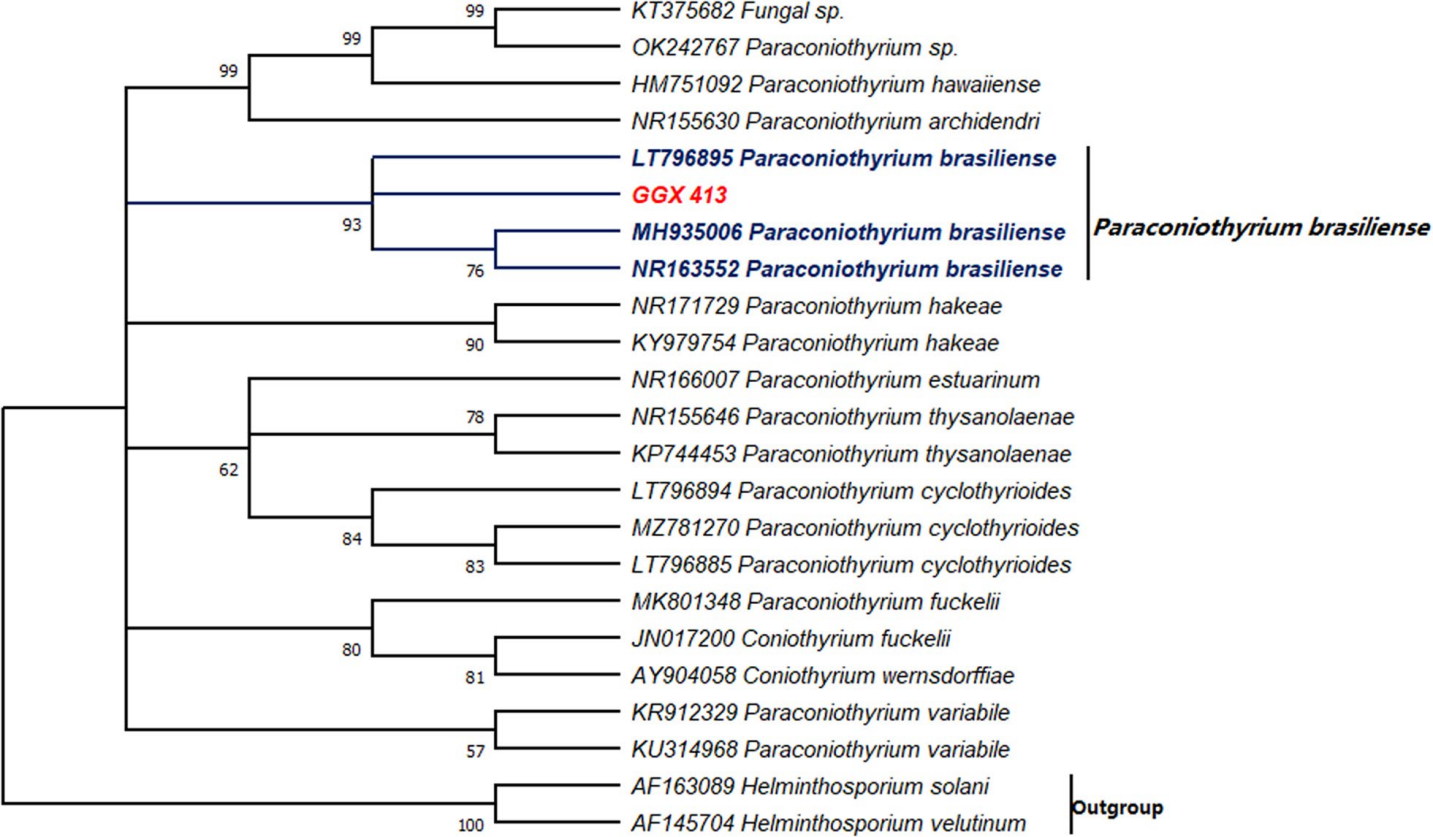
Phylogenetic tree was constructed based on ITS sequences; bootstrap values (1000 replicates) > 50% are shown at the nodes. Sequences of *Helminthosporium velutinum* and *H. solani* were used as the outgroup

An ITS sequence with a length of 552 bp was obtained and aligned with sequences from the GenBank database using the BLASTN algorithm to identify the fungal species. Based on the alignment, representative sequences with similarity to GGX 413 of > 90% were used in a phylogenetic analysis, and the ITS sequences of *Helminthosporium velutinum* (AF145704) and *H. solani* (AF163089) were used as the outgroup. As shown in Fig. 2, GGX 413 was found to belong to the *Paraconiothyrium brasiliense* group, with a bootstrap value of 93%, indicating that the evolutionary relationship was reliable.

**Fig. 3:**
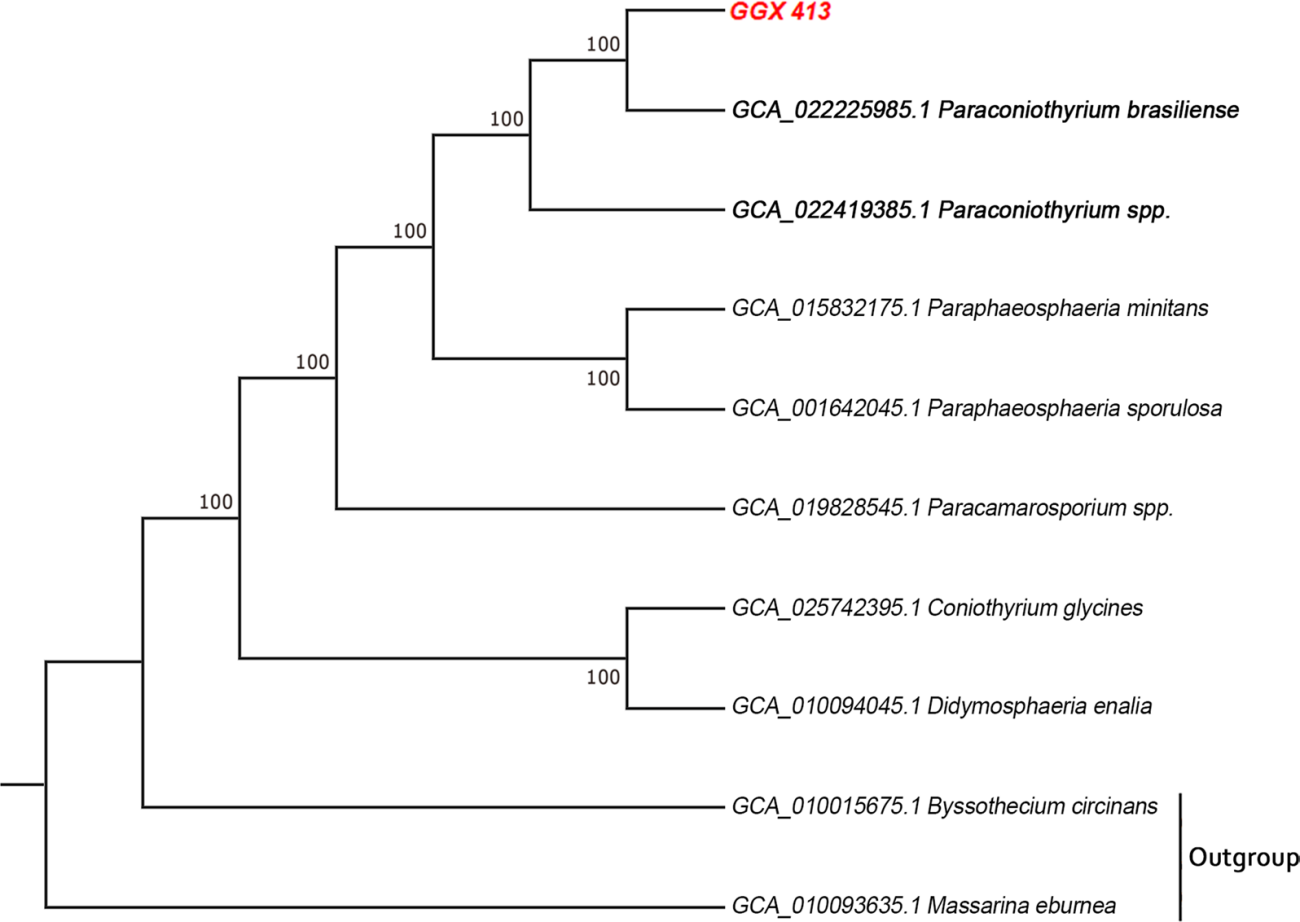
Phylogenetic tree of single-copy gene families shared by 10 species was generated with the maximum likelihood method, with 100 bootstrap replications. *Massarina eburnea* and *Byssothecium circinans* were used as the outgroups

A phylogenetic tree was also generated based on the 4995 single-copy gene families shared by 10 species, to determine their genomic similarity to GGX 413. As shown in Fig. 3, GGX 413 is closely related to *P. brasiliense* M42-189, isolated from Apricot trees, and to *Paraconiothyrium* sp. Pb-2020, isolated from the Chinese white wax scale insect.

### Sequence assembly and genomic characteristics

In total, 11 scaffolds were obtained by assembling the sequence reads, and a genome sequence of 39.49 Mb with a GC content of 51.2% was generated. The minimum and maximum sequence lengths were 0.24 Mb and 5.24 Mb, respectively, and the length of the N50 fragment was 4.62 Mb. The prediction of protein-coding genes showed that the total length of the protein-coding genome was 20.17 Mb, containing 13,057 genes and accounting for 51.1% of the total genome length. The statistical details of gene assembly are shown in Table 1.

**Table 1:** Scaffolds and genomic features of *Paraconiothyrium brasiliense* GGX 413

Features	Value
*Scaffolds*	
Total sequenced length (bp)	39,490,049
Total sequence number	11
Min sequence length (bp)	237,784
Max sequence length (bp)	5,242,516
N20	5,177,266
N50	4,615,948
N90	2,343,146
GC content %	51.2
*Genome of protein-coding*	
Total genes length (bp)	20,170,337
Genes percentage of genome	51.1%
Total genes number	13,057
Average gene length (bp)	1544.7
Total exons number	33,954
Average exons per gene	2.6
Total exons length (bp)	18,702,916
Exons percentage of genome	47.36%
Average exons length (bp)	550.8
Average introns length (bp)	70.2
Total CDSs length (bp)	18,702,916
CDSs percentage of genome	47.36%
Average CDS length (bp)	1432.4

**Fig. 4:**
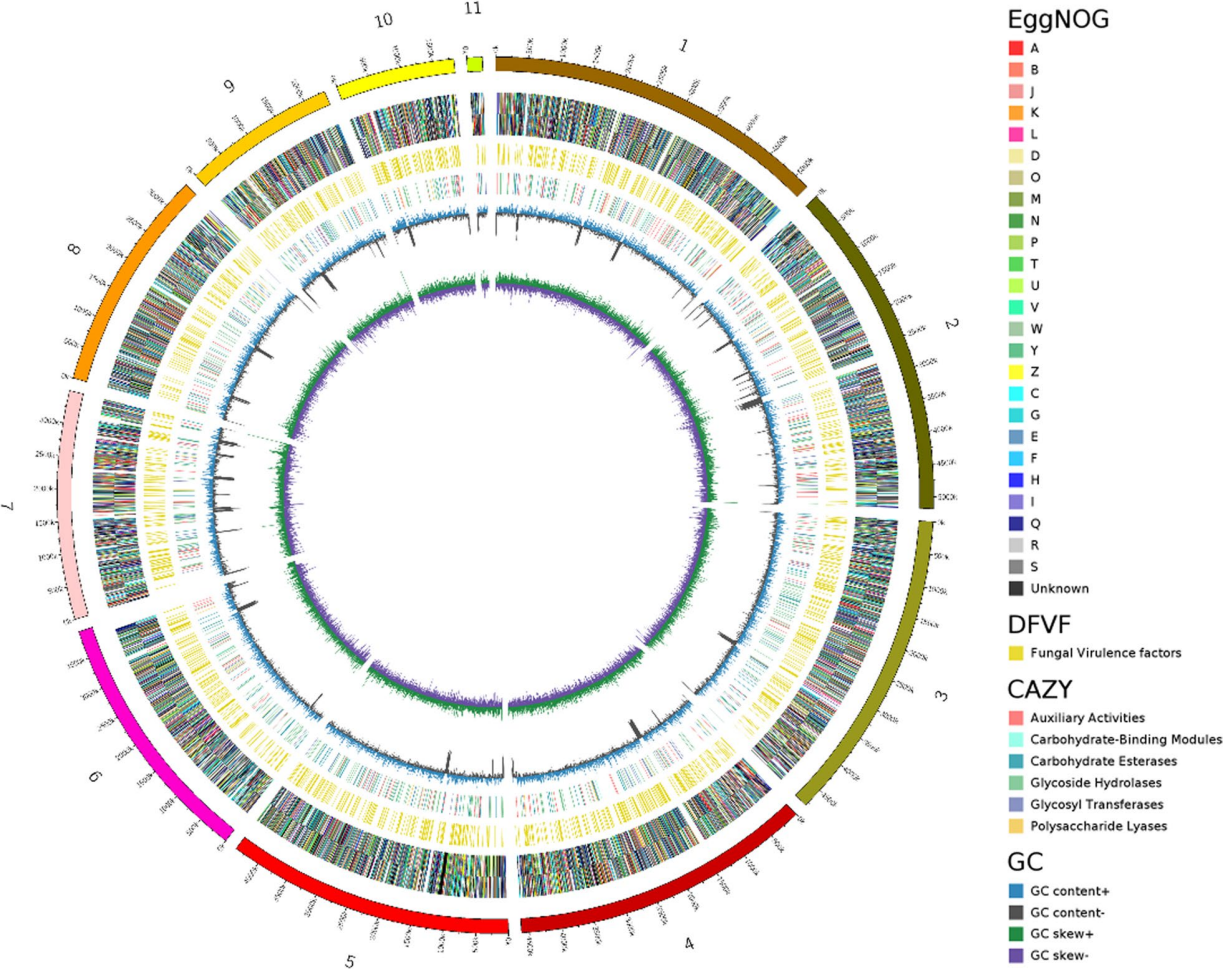
Circular diagram of the *P. brasiliense* GGX 413 genome

The detailed characteristics of the whole *P. brasiliense* GGX 413 genome are shown in a circular diagram (Fig. 4). From the inner circle to the outer circle, the corresponding characteristics are the GC skew (green > 0, purple < 0), GC content (blue > mean, gray < mean), analysis of CAZymes, DFVF, KOG functional classification in negative chain genes, and KOG functional classification in positive chain genes.

### Predicted repeat sequences

In total, 332,704 bp of repeat sequences were identified, accounting for 0.84% of the whole genome, of which 0.82% (of the whole genome) was interspersed repeats and 0.02% was tandem repeats. Among the interspersed repeats, the total numbers of retroelements and DNA transposons were 1175 and 421, respectively, corresponding to 0.72% and 0.09% of the genomic length, respectively. The retroelements were mainly classifiable into four groups: long interspersed nuclear elements (LINEs), short interspersed nuclear elements (SINEs), long terminal repeat (LTR), and Penelope retroelements. Gypsy/DIRS1, in the LTR group, was the dominant retroelement, accounting for 0.6% of the whole genome. Hobo-Activator was the dominant DNA transposon, accounting for 0.02% of the genomic length. The tandem repeat sequences mainly consisted of satellites, simple repeats, and low-complexity repeats. The statistical analysis of the repeat sequences is shown in Table 2.

**Table 2:** Statistical analysis of repeat sequences in the GGX 413 genome

Repeat type	Number of elements	Length occupied (bp)	Percentage of genome (%)
*Interspersed repeats*			
Retroelements	1175	282,872	0.72
SINEs	1	95	0.00
LINEs	255	17,693	0.04
CRE/SLACS	5	295	0.00
L2/CR1/Rex	36	2316	0.01
R1/LOA/Jockey	55	3982	0.01
R2/R4/NeSL	10	645	0.00
RTE/Bov-B	18	1129	0.00
L1/CIN4	40	2578	0.01
LTR elements	919	265,084	0.67
BEL/Pao	64	4798	0.01
Ty1/Copia	189	17,195	0.04
Gypsy/DIRS1	597	238,312	0.60
Retroviral	53	3409	0.01
Penelope	22	1763	0.00
DNA transposons	421	35,104	0.09
hobo-Activator	106	8053	0.02
Tc1-IS630-Pogo	37	3903	0.01
PiggyBac	8	505	0.00
Tourist/Harbinger	21	1727	0.00
Unclassified	69	6500	0.02
*Tandem repeats*			
Satellites	49	5002	0.01
Simple repeats	41	4256	0.01
Low complexity	4	449	0.00

### Predicted ncRNAs

A total of 181 ncRNAs were predicted, including 43 rRNAs, 105 tRNAs, and 33 other ncRNAs. The average and total lengths of the ncRNAs were 514.2 bp and 93,073 bp, respectively, accounting for 0.2353% of the whole genome. The ncRNA with the highest copy number was tRNA, accounting for 0.0253% of the whole genome. rRNA consisted mainly of 5S rRNA, 5.8S rRNA, 18S rRNA, and 28S rRNA, and the latter three had the highest numbers of gene copies. The ncRNAs identified in the GGX 413 genome are shown in Table 3.

**Table 3:** Statistical analysis of ncRNA in the GGX 413 genome

Type	Copy	Average length (bp)	Total length (bp)	Percentage in genome (%)
5S rRNA	1	115.0	115	0.0002
5.8S rRNA	14	151.8	2125	0.0053
18S rRNA	14	1796.7	25,154	0.0636
28S rRNA	14	3652.4	51,133	0.1294
tRNA	105	95.2	9995	0.0253
oncRNA	33	137.9	4551	0.0115

### Predicted pathogenicity-related genes

**Fig. 5:**
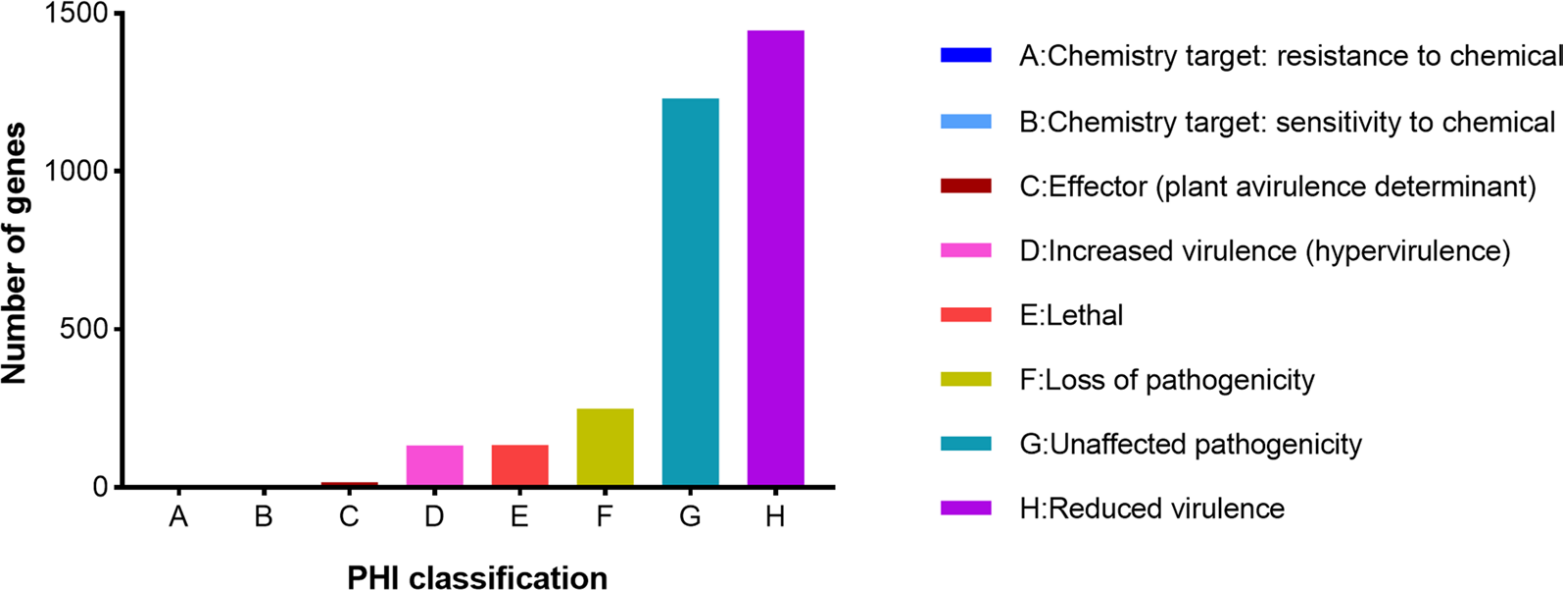
PHI functional classification of proteins encoded in the GGX 413 genome

A whole-proteome BLAST search against the PHI database predicted a total of 2905 (22.2%) genes, mainly classified into eight groups. As shown in Fig. 5, 1579 genes were associated with virulence: 1446 were associated with reduced virulence and 133 genes were associated with hypervirulence. Eleven genes were involved in chemical susceptibility: four resistance genes and seven sensitivity genes. The remaining genes were mainly distributed in “unaffected pathogenicity” (1230), “loss of pathogenicity” (249), and “lethal” (134).

**Fig. 6:**
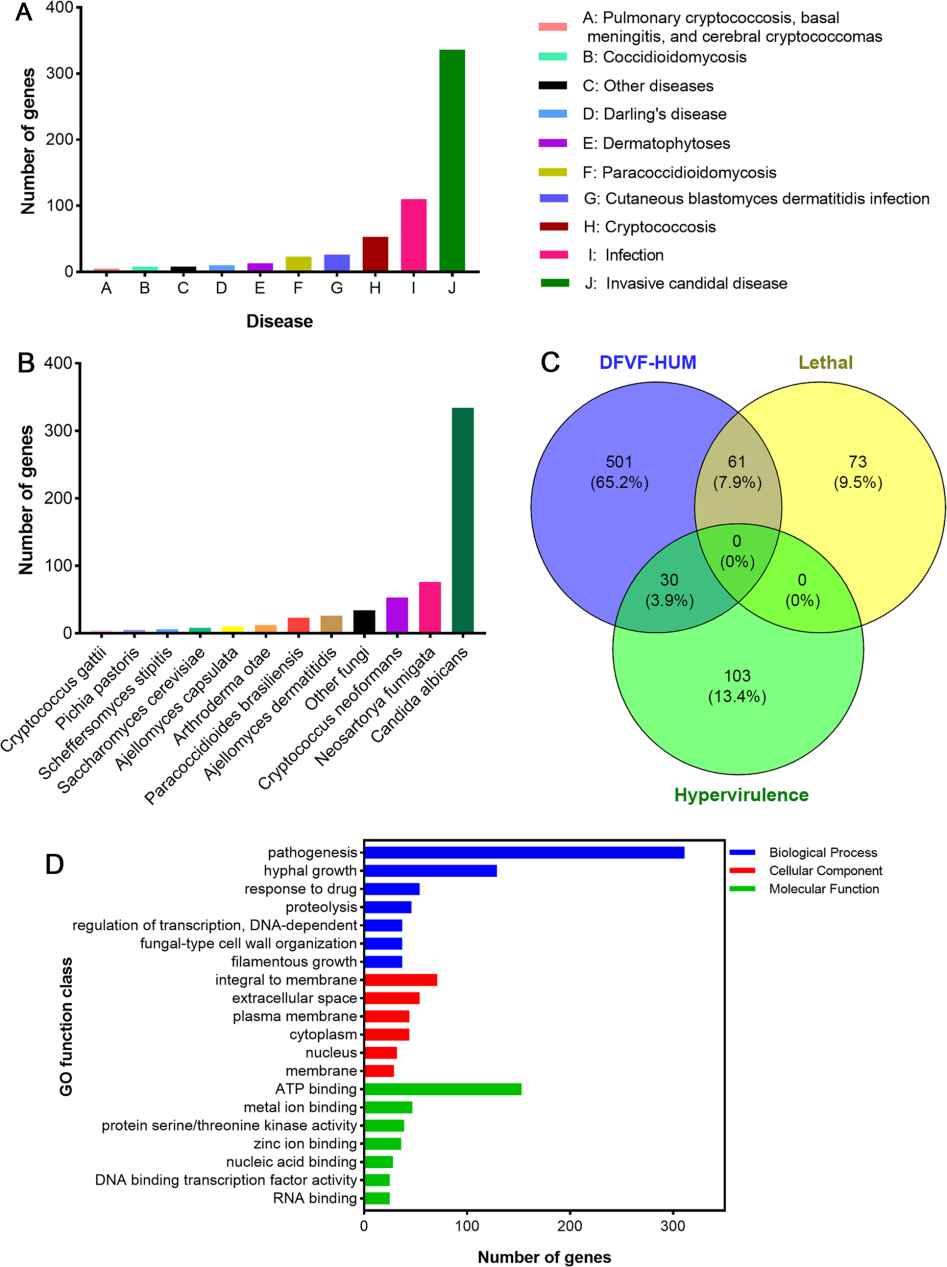
Analysis of genes encoding DFVF-HUM in the GGX 413 genome. **A** Genes predicted to encode DFVF-HUM. **B** Organismal sources of genes encoding DFVF-HUM. **C** Venn diagrams of genes encoding DFVF-HUM, lethal virulence factors, and hypervirulence factors. **D** GO functional annotation of genes encoding DFVF-HUM

Virulence factor-related genes were also predicted with DFVF, and a total of 1186 (9.08%) genes were associated with disease: 592 genes were involved in human disease, and 594 genes were involved in animal or plant disease. Most of the genes related to human disease were associated with invasive candidal disease (336), mainly caused by *Candida albicans*, followed by infection (110) and cryptococcosis (53), mainly caused by *Neosartorya fumigata* and *Cryptococcus neoformans*, respectively (Fig. 6A and B). Combined with the prediction data from the PHI database, we screened for genes encoding highly virulent or lethal virulence factors that could cause disease in humans. As shown in Fig. 6C, 61 of the 592 genes involved in human disease encoded lethal virulence factors, and 30 of them encoded hypervirulence factors. The GO functional annotation of the genes encoding virulence factors associated with human diseases (DFVF-HUM) was also analyzed (Fig. 6D): 525 genes were involved in “molecular function,” followed by “biological process” (483) and “cellular component” (370). These genes were mainly distributed in “pathogenesis” (311), “ATP binding” (153), and “hyphal growth” (129).

### KOG functional classification of proteins encoded in the *P. brasiliense* GGX 413 genome

**Fig. 7:**
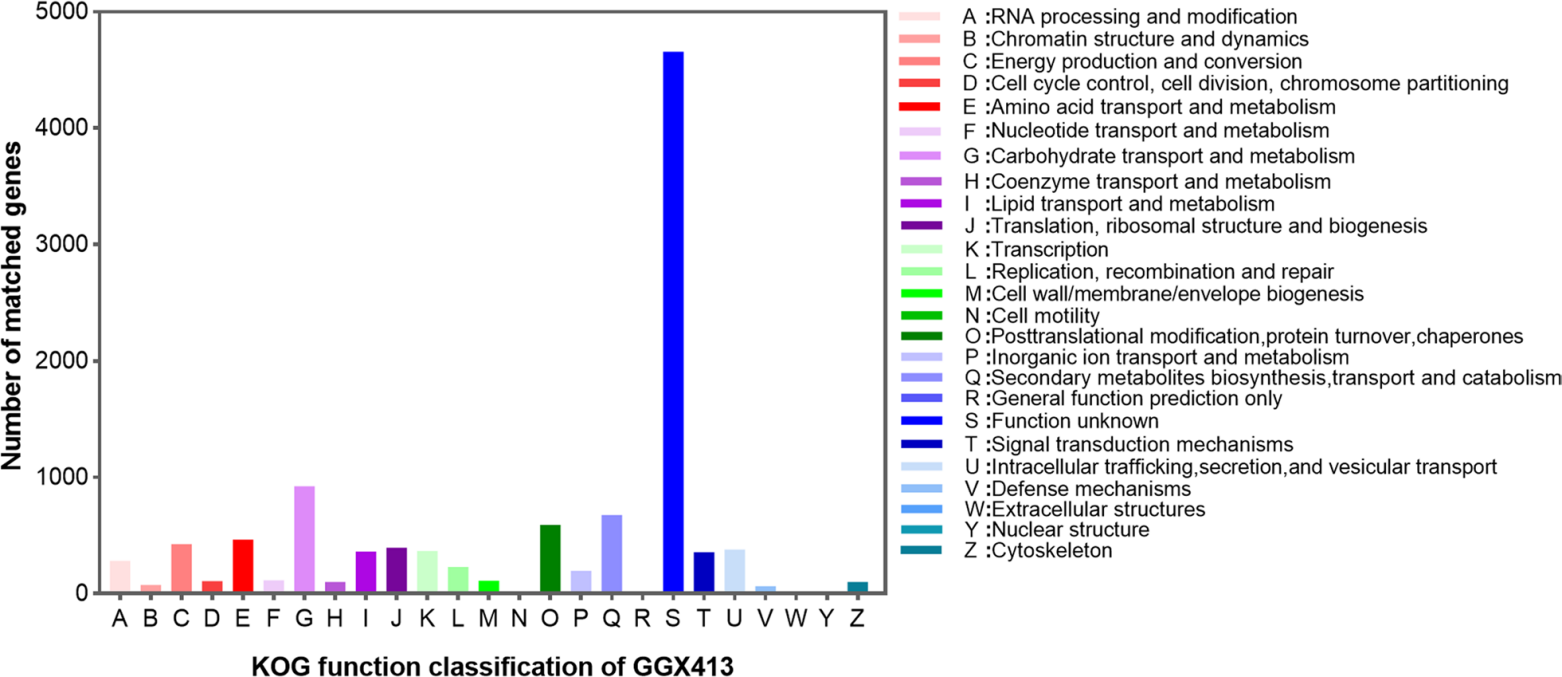
KOG functional classification of proteins encoded in the GGX 413 genome

Eleven thousand genes were classified into protein functional categories based on a KOG analysis and accounted for 84.2% of the total number of ORFs in the *P. brasiliense* GGX 413 genome. Except for class S genes of function unknown (4658), the class G genes, involved in carbohydrate transport and metabolism, had the highest number of genes (925), followed by the class Q genes, which are related to secondary metabolite biosynthesis, transport, and catabolism (677), the class O genes, which are involved in posttranslational modification, protein turnover, and chaperones (590), and the class E genes, which are related to amino acid transport and metabolism (463) (Fig. 7). Combined with the virulence factor data on human diseases, 27 genes encoding DFVF-HUM were involved in the synthesis of secondary metabolites.

### KEGG functional annotation of *P. brasiliense* GGX 413 genes

**Fig. 8:**
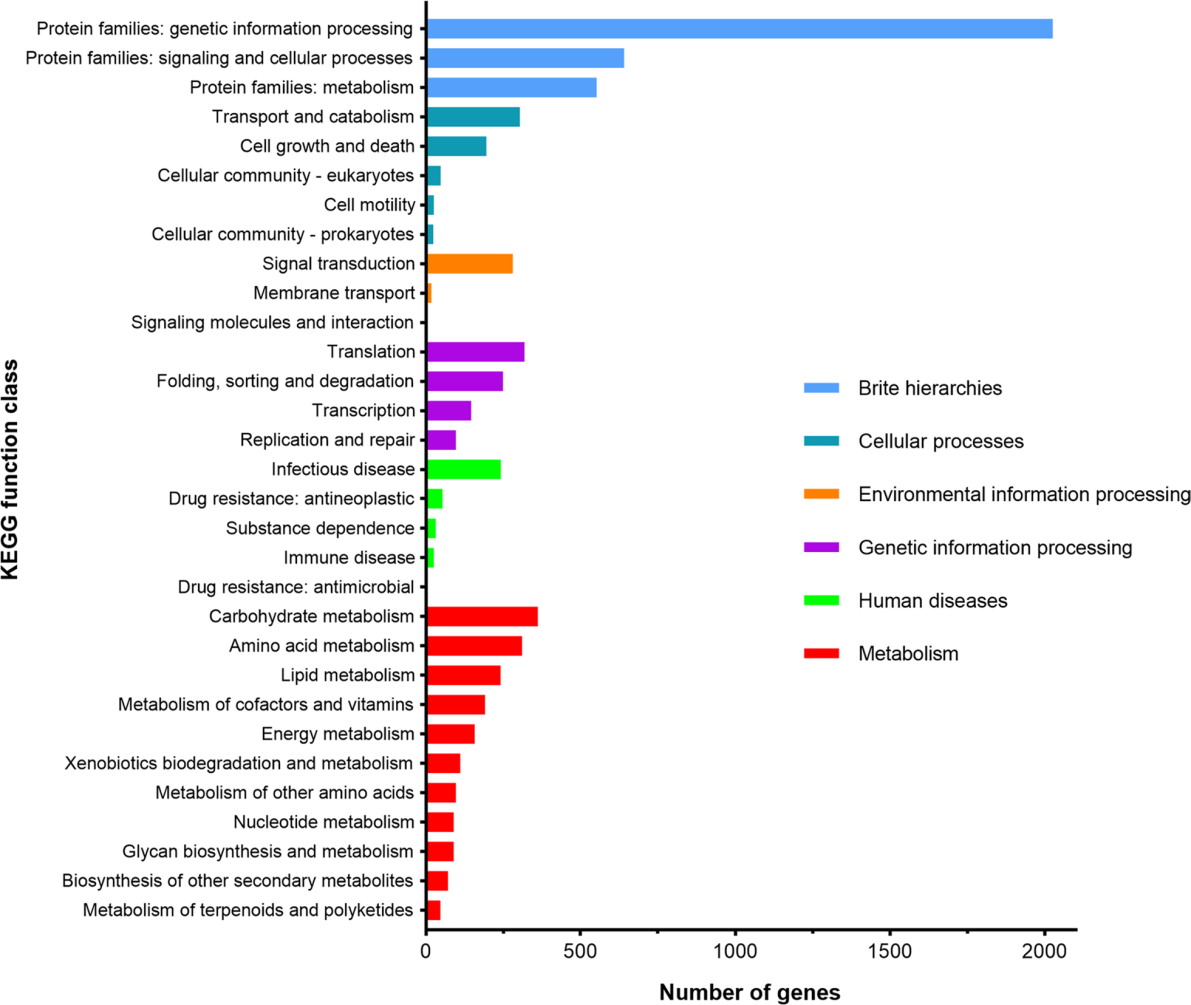
KEGG functional annotation of GGX 413 genes

A KEGG functional analysis was performed to better understand the functions encoded in the *P. brasiliense* GGX 413 genome (Fig. 8). In total, 4104 genes were annotated with the KEGG database, accounting for 31.4% of the total number of ORFs in the GGX 413 genome, and were involved in 462 KEGG pathways (Additional file 1: Table S1). The classes with the greatest enrichment were “BRITE hierarchies” (3219), including subclasses “protein families: genetic information processing” (2026), “protein families: signaling and cellular processes” (641), and “protein families: metabolism” (552), which were also the top three gene-rich subclasses in the KEGG pathway groupings. The class with the second greatest number of genes was “metabolic” (1770), followed by “human disease” (871). In the class “human disease,” 242 genes assigned to 27 KEGG pathways were involved in human infectious diseases, and the pathway with the greatest gene enrichment was KO: 05131 (name: *Shigellosis*), followed by KO: 05165 (name: *Human papillomavirus* infection) and KO:05166 (name: *Human T-cell leukemia virus 1* infection). We also concluded that the genes encoding mitogen-activated protein (MAP) kinase (MAPK), p38 MAPK, and cytochrome c were associated with 17, 15, and 14 human infectious disease pathways, respectively (Table 4). In the class “metabolic,” 119 genes were associated with secondary metabolites, 47 of which were involved in the metabolism of terpenoids and polyketides. A further analysis was performed combining the virulence factor data on human diseases, which showed that four genes encoding DFVF-HUM were involved in terpenoid metabolism. The proteins encoded by two of these four genes were acetyl-CoA C-acetyltransferases, and the other two genes encoded mevalonate kinase and squalene monooxygenase.

**Table 4:** Analysis of human infectious disease KEGG pathways for *P. brasiliense* GGX 413

Ranking of the number of genes involved in infectious disease pathways		Ranking of the number of pathways involved by a single gene	
KEGG pathway	Number of genes	Gene product	Number of pathways
KO: 05131	46	MAPK	17
KO: 05165	44	p38	15
KO:05166	42	CYC	14
KO:05170	32	RAC1	10
KO:05130	29	MAP2K1	9
KO:05169	28	RHOA	7
KO:05152	23	CDC42	7
KO:05163	23	PRKCA	7
KO:05110	21	GSK3B	7
KO:05164	20	KRAS	7

### GO functional annotation of *P. brasiliense* GGX 413 genes

**Fig. 9:**
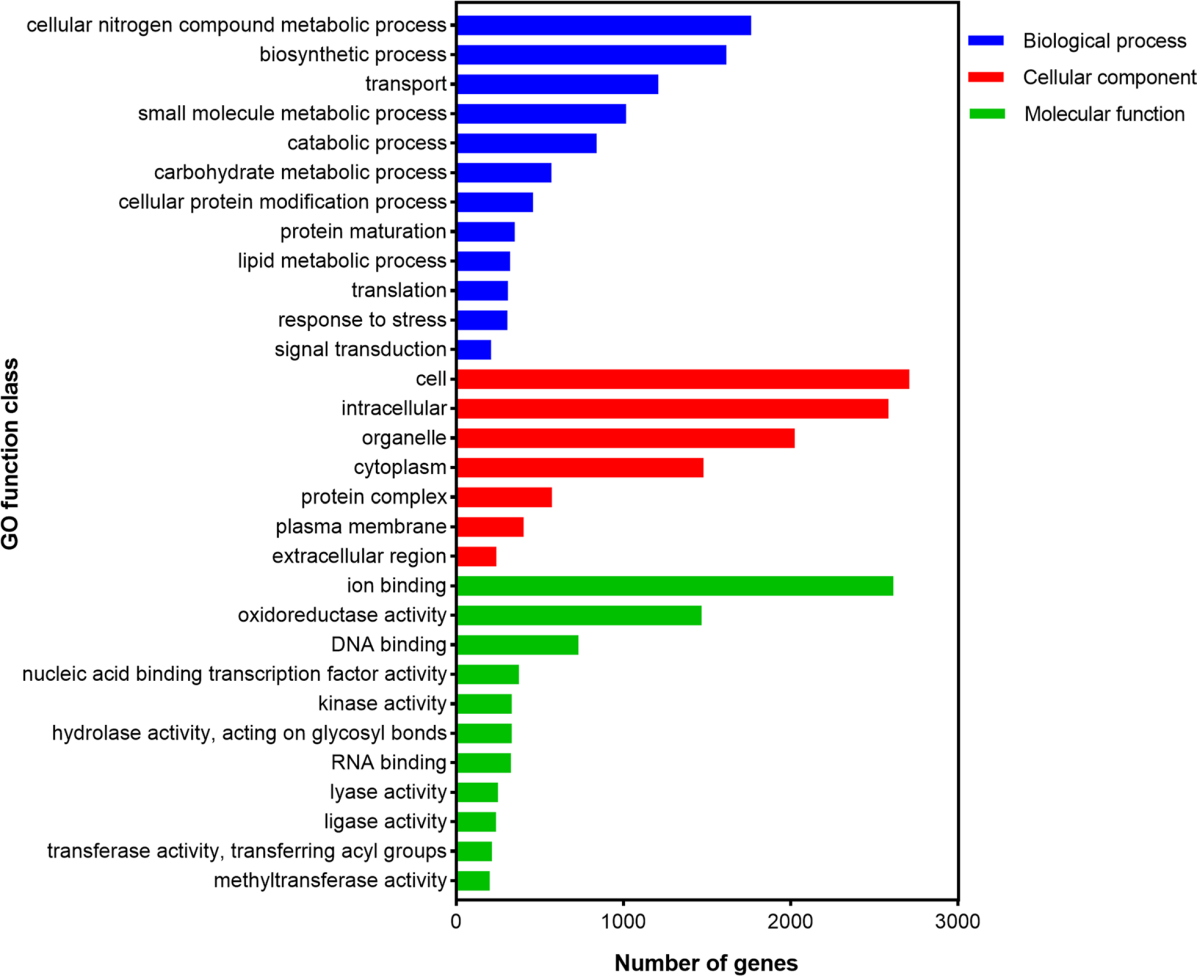
GO functional annotation of GGX 413 genes

The GO annotation of *P. brasiliense* GGX 413 genes was performed with InterProScan; 7801 proteins were identified and the corresponding GO numbers were obtained. According to the analysis in the Generic GO-Slim database, the GO terms were divided into three major groups: “biological process,” “cellular component,” and “molecular function.” Among these, 7136 genes were involved in “biological process,” followed by “molecular function” (6773) and “cellular component” (3898). As shown in Fig. 9, the genes were mainly enriched in “cell” (2708), “ion binding” (2612), “intracellular” (2583), “organelle” (2022), and “cellular nitrogen compound metabolic process” (1763).

### Determination of secretome and secretory peptidases associated with human diseases

**Fig. 10:**
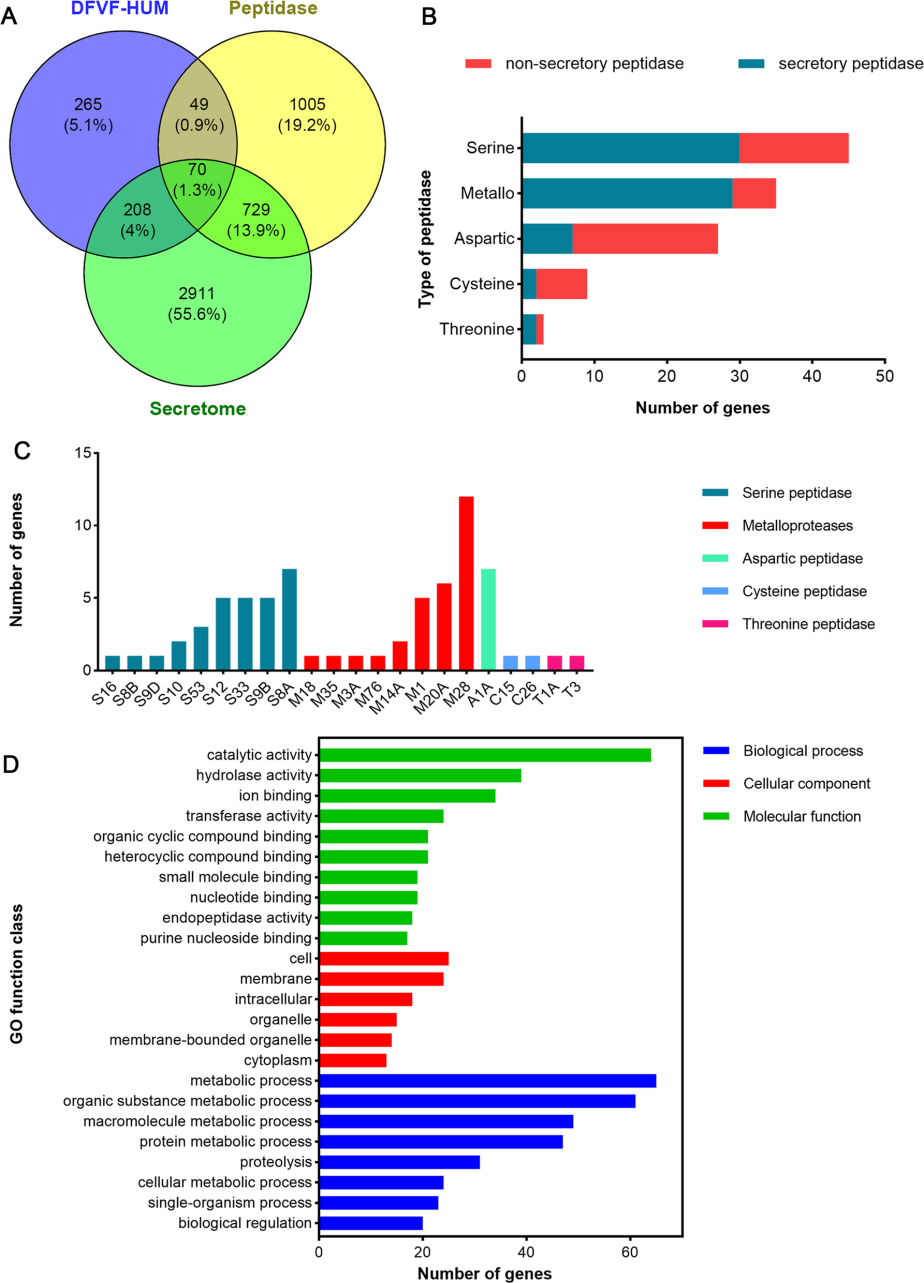
Analysis of genes encoding secretory peptidases associated with human diseases in the GGX 413 genome. **A** Venn diagrams of genes encoding DFVF-HUM, peptidase, or secretome proteins. **B** Numbers of genes associated with different types of peptidases involved in human diseases. **C** Numbers of genes associated with different types of secretory peptidases related to human diseases. **D** GO functional annotation of genes encoding secretory peptidases associated with human diseases

The predicted secretome showed that a total of 841 proteins encoded by 3918 genes were secretory proteins (Additional file 2: Table S2), and 92 of them encoded by 278 genes were associated with human disease. Peptidase-related genes were also predicted with the MEROPS database, and a total of 1853 genes were involved in peptidase synthesis. To screen for secretory peptidases associated with human pathogenicity, the gene profiles of the secretome, peptidase, and DFVF-HUM were compared. As shown in Fig. 10A, 119 genes were shared between peptidases and DFVF-HUM, and 70 genes among the secretome, peptidases, and DFVF-HUM, which suggests that peptidases encoded by 119 genes are involved in human disease and 70 of them were secreted. These peptidases were divided into five categories according to their catalytic type, including serine peptidases, metallopeptidases, aspartic peptidases, cysteine peptidases, and threonine peptidases. The main secretory peptidases were serine peptidase and metallopeptidase (Fig. 10B), and the most abundant serine peptidase and metallopeptidase were the subtilisin subfamily (8A) and the aminopeptidase Y (M28) family, respectively (Fig. 10C). The GO functional annotation of the 70 genes encoding the secretory peptidases associated with human diseases was also analyzed. All of the genes were involved in “molecular function” and “biological process,” but only 46 genes were associated with “cellular component.” The analysis of “molecular function” showed that peptidases encoded by 70 genes play important roles in catalytic, hydrolase, and ion binding (Fig. 10D).

### Analysis of CAZymes

In total, 826 genes encoding CAZymes and 15 genes encoding carbohydrate-binding modules (CBMs) were identified with the CAZy database, accounting for 6.44% of the total number of open reading frames (ORFs) in the *P. brasiliense* GGX 413 genome. With a sequence-based classification, the CAZyme-encoding genes fell into 174 CAZy protein families and the CBM-encoding genes fell into eight families. Glycoside hydrolases (GHs), which catalyze the hydrolytic cleavage of glycosidic bonds to generate the carbohydrate hemiacetal, had the greatest number of genes, which were mainly distributed in the GH16 (21), GH3 (20), GH109 (17), and GH28 (15) families. One hundred seventy-seven genes encoded auxiliary activities (AAs), which are redox enzymes that act in conjunction with CAZymes, and these were mainly distributed in the AA7 (56) and AA9 (39) families. The genome contained 176 genes encoding carbohydrate esterases (CEs), mainly distributed in the CE10 (82) and CE1 (32) families, which catalyze the de-*O*-acetylation or de-*N*-acylation of substituted saccharides. The number of genes encoding glycosyl transferases (GTs), polysaccharide lyases (PLs), and CBMs was 94, 30, and 15, respectively. The relevant statistical data are presented in Table 5.

**Table 5:** Statistical analysis of CAZy

Type of CAZy	Number of Genes	Percentage (%)	Number of families
GT	94	0.72	29
PL	30	0.23	15
CE	176	1.35	12
AA	177	1.36	19
GH	349	2.67	99
CBM	15	0.11	8

**Fig. 11:**
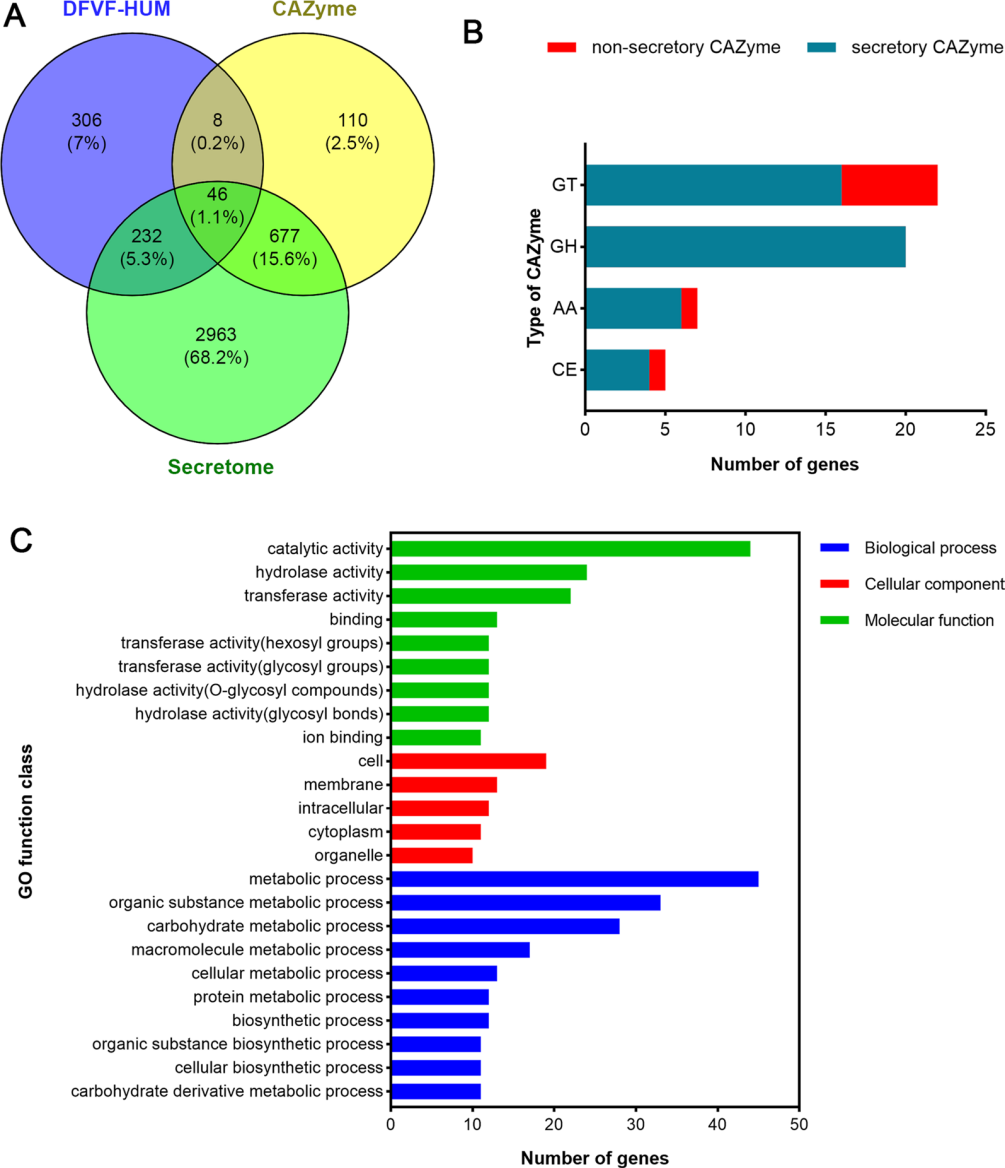
Analysis of genes encoding secretory CAZymes associated with human diseases in the GGX 413 genome. **A** Venn diagram of genes encoding DFVF-HUM, CAZymes, and secretome. **B** Numbers of genes for different types of CAZymes involved in human diseases. **C** GO functional annotation of genes encoding secretory CAZymes associated with human diseases

The secretory CAZymes associated with human pathogenicity were also screened by comparing the gene profiles of the secretome, CAZymes, and DFVF-HUM. Venn diagram shows that 54 genes were shared between CAZymes and DFVF-HUM, and 46 genes were shared among the secretome, CAZymes, and DFVF-HUM, which suggests that CAZymes encoded by 54 genes contribute to human diseases and 46 of them have secretory properties (Fig. 11A). The secretory CAZymes associated with human pathogenicity were mainly distributed in the GT, GH, AA, and CE families (Fig. 11B), and a GO functional analysis showed that these CAZymes play crucial roles in transferring hexosyl and glycosyl groups, hydrolyzing O-glycosyl compounds, and ion binding (Fig. 11C).

### Prediction of secondary metabolite biosynthetic gene clusters

**Fig. 12:**
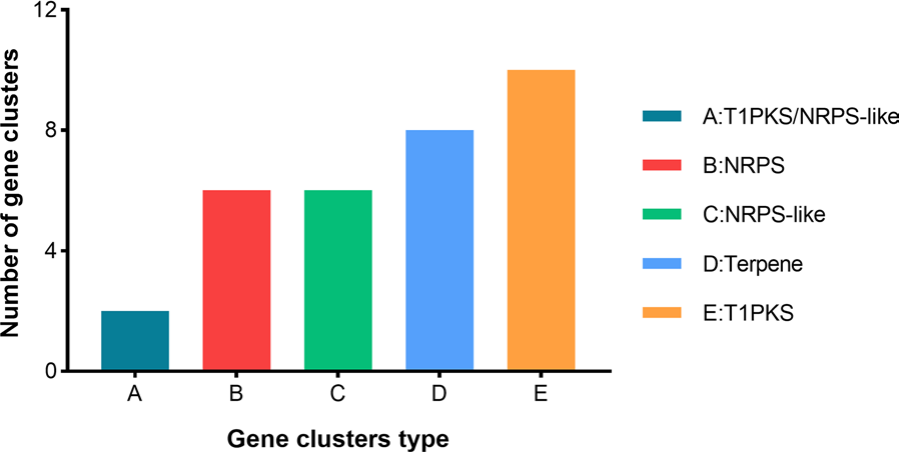
Analysis of secondary metabolite biosynthetic gene clusters

Filamentous fungi produce various bioactive secondary metabolites, which have been exploited for the development of drugs. The genes encoding secondary metabolites tend to aggregate in biosynthetic gene clusters. The analysis of secondary metabolite gene cluster with the antiSMASH tool identified a total of 32 gene clusters of secondary metabolites, distributed into five types. As shown in Fig. 12, type I polyketide synthase (T1PKS) had the highest number of gene clusters (10), followed by terpenes (8), nonribosomal peptide synthetase clusters (NRPS) (6), NRPS-like clusters (6), and T1PKS/NRPS-like clusters (2). The similarity between the five gene clusters and known gene clusters was 100%, and the products encoded by the known gene clusters were melanin, dimethylcoprogen, ( −)-mellein, and clavaric acid.

### Genome specificity analysis of *P. brasiliense* GGX 413

**Fig. 13:**
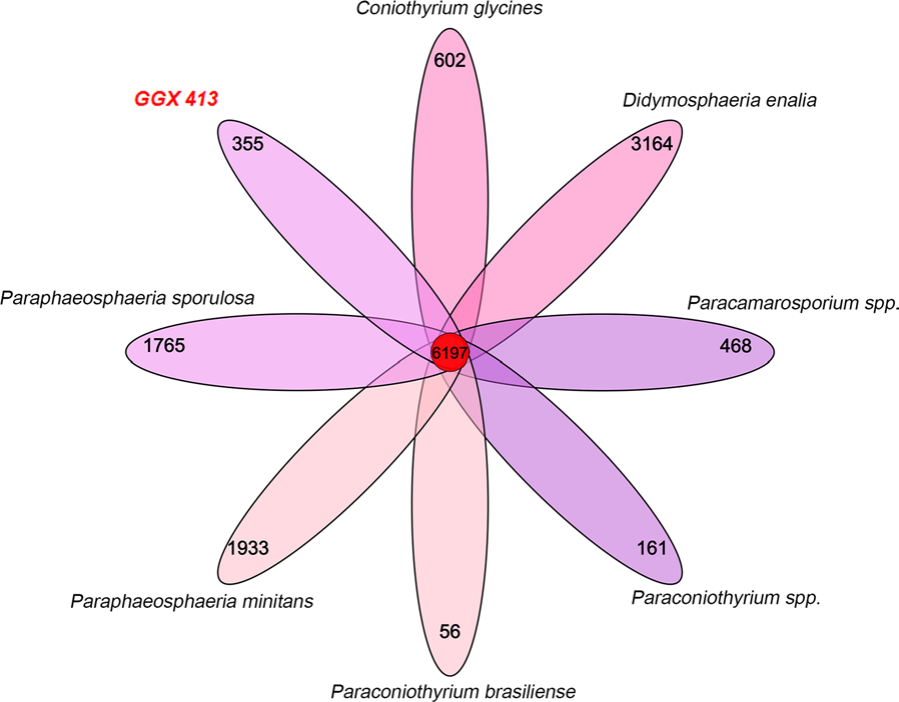
Analysis of specific gene families of *P. brasiliense* GGX 413

**Fig. 14:**
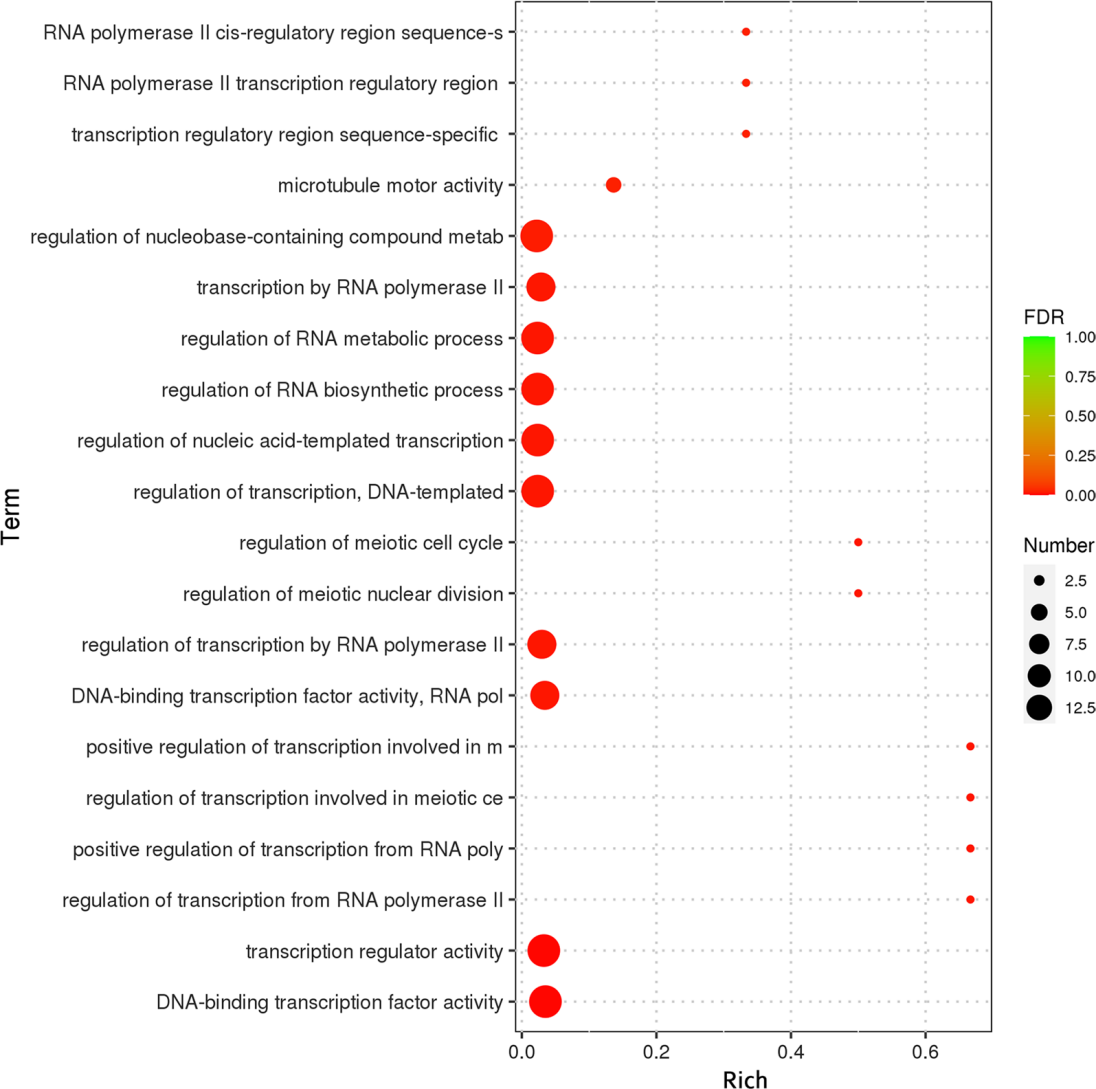
GO enrichment analysis of specific genes in the GGX 413 genome

The specific genes of *P. brasiliense* GGX 413 were identified by analyzing the gene families with seven closely related strains, and enrichment analyses of their GO function were performed. As shown in Fig. 13, the 357 specific genes of *P. brasiliense* GGX 413 were assigned to 355 gene families, and two of them contained four genes. The GO enrichment analysis showed that the specific genes of GGX 413 were mainly related to the regulation of RNA biosynthesis and metabolic process (Fig. 14).

**Fig. 15:**
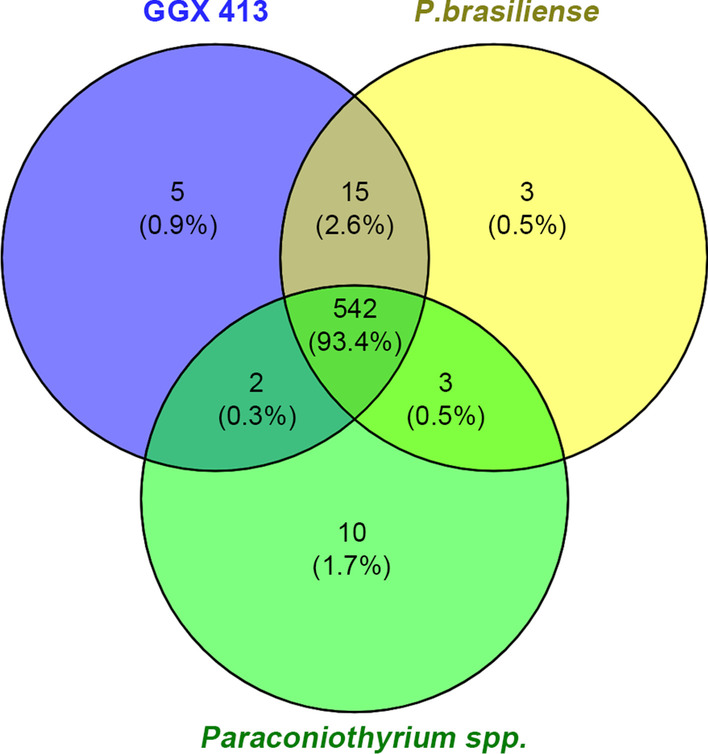
Analysis of the specific virulence factors of *P. brasiliense* GGX 413

The specificity of *P. brasiliense* GGX 413 virulence factors was also analyzed. Compared with the virulence factors of *P. brasiliense* M42-189 and *Paraconiothyrium* sp. Pb-2020, five specific virulence factors were detected (Uniprot ID: Q59RW3_CANAL, A6ZLA2_YEAS7, CARP3_CANAL, STE20_USTMA and CUTI_ERYGR), and three of them (Q59RW3_CANAL, CARP3_CANAL and A6ZLA2_YEAS7) were involved in infectious diseases in humans (Fig. 15).

## Discussion

The ITS region of ribosomal RNAs, which lies between the small subunit ribosomal RNA gene and the large subunit ribosomal RNA gene, is a widely used phylogenetic marker for fungi [12]. Therefore, we used the ITS sequence to identify the species of the isolated strain. Based on the BLASTN algorithm and a phylogenetic analysis, the isolated strain was identified as *P. brasiliense*. Single-copy gene families, which are characterized by uniqueness, high sequence conservation across species, and biparental inheritance, have long been recognized as ideal molecular markers with which to infer relationships of closely related species [13]. Similarly, the phylogenomic analysis of single-copy gene families in this study also confirmed the close relationship between the isolated strain and *P. brasiliense.*

According to a virulence factor analysis using the DFVF database, 56.8% of the 592 virulence genes of *P. brasiliense* GGX 413 involved in human diseases were associated with invasive candidal disease, mainly caused by *Candida albicans.* It has been reported that 10% to 45% of fungal ocular infections are caused by *C. albicans*, and the expression of genes associated with adhesion to the substratum, efflux pumps, transcription factors, and virulence are upregulated during infection [14]. Therefore, understanding the pathogenic mechanisms of ocular infections caused by *C. albicans* provides a good basis for examining the pathogenesis of human eyelid cellulitis caused by *P. brasiliense.* 66 of the 592 virulence genes were derived from biphasic fungi (including *Ajellomyces capsulata*, *A. dermatitidis*, *Coccidioides*, *Paracoccidioides brasiliensis*, and *Penicillium marneffei*). The source of 53 of them was *Cryptococcus neoformans*, and the source of 13 was *Arthroderma*. These fungi are highly pathogenic to humans and cause infections of human skin tissue. Lethal and hypervirulence genes can be retrieved from PHI database and can be used to design a new generation of genomic-based fungicides [15]. Therefore, 61 genes encoding lethal virulence factors and 30 genes encoding hypervirulence factors identified by us can be used as targets for fungal treatments in the future.

To comprehensively understand the gene distribution characteristics of *P. brasiliense* GGX 413, we systematically analyzed the genetic data with KOG, KEGG, and GO functional annotations. The molecular biological functions and functional classification of the genes thus obtained extend our understanding of the pathogenic mechanism of this strain in humans. As well as virulence factors, secondary metabolites play an important role in fungal pathogenicity in humans [16]. *Paraconiothyrium brasiliense* produces various secondary metabolites, including terpenes, polyketides, and aromatic compounds that have a wide range of biological activities [17]. In this study, we identified 27 genes encoding DFVF-HUM involved in secondary metabolite synthesis and four genes related to terpenoid metabolism, which were associated with human disease. It has been reported that melanin protects fungal hyphae from clearance by the immune system through the establishment of a niche within phagocytes [18]. According to our analysis of secondary biosynthetic gene clusters, two T1PKS gene clusters were involved in melanin production. In the pathogenic processes of fungi, MAPK pathways play an important role in maintaining cellular integrity, morphogenesis and cell wall formation, invasive growth under embedded conditions, and biofilm formation [19]. p38 MAPK, one of the MAPK family members, has been shown to be associated with the oxidative stress caused by the overproduction of reactive oxygen species, which is recognized as an important factor in ocular surface diseases [20]. To survive within the human host, pathogenic fungi must protect themselves from oxidative stress, and cytochrome c contributes to their resistance to it [21]. In this study, we found that the genes encoding MAPK, p38, and CYC were involved in the largest number of human infectious disease pathways, including pathways with the greatest gene enrichment (KO: 05131, KO: 05165, KO:05166, and KO:05170). Therefore, these genes can be used as potential targets for the treatment of *P. brasiliense* GGX 413 infections.

Peptidases, which are also described as proteases, proteinases, and proteolytic enzymes, cleave specific peptide bonds in various protein substrates. The secretory peptidases are not only involved in fungal growth, but are also related to pathogenesis of fungi. The secretory serine proteinases contributed to the pathogenesis of *Epicoccum purpurescens* by inducing allergy and inflammation [22]. The secretory metalloproteases are potential virulence factors during host invasion by *Trichophyton mentagrophytes* [23]. The best-characterized secretory enzymes implicated in human diseases are serine proteases of the subtilisin subfamily (8A), metalloproteases of the deuterolysin (M35) and fungalysin (M36) families, and aspartic proteases of the pepsin family (A1) [24]. We have shown that the most common secretory peptidases of *P. brasiliense* GGX 413 are serine peptidases of the subtilisin subfamily (8A) and metallopeptidases of the aminopeptidase Y (M28) family, possibly caused by strain differences. GO functional annotation indicated that the major roles of these secretory peptidases are catalytic, hydrolysis, and ion binding. Pathogenic fungi can directly damage host cells by secreting hydrolytic enzymes or lytic toxins in the form of peptides or small metabolites. Iron is an essential metal in the pathogenic process of fungi, and reductive iron uptake is associated with the virulence of fungi and with morphogenesis in dimorphic or pleiomorphic fungi [25].

Carbohydrate-active enzymes are very important for the survival and pathogenicity of fungi because they are responsible for breaking down the components of the host to permit the acquisition of nutrients and the establishment of a successful infection process [26]. Therefore, the prediction of genes encoding CAZymes is essential to understanding the pathogenesis of *P. brasiliense* GGX 413 in humans. It has also been reported that some virulence genes associated with carbohydrate metabolism are involved in horizontal gene transfer, which could cause a sudden outbreak of very highly virulent fungus [27]. With a comprehensive analysis, we predicted that the CAZymes encoded by 54 virulence genes were associated with human diseases, and 85% of them encoded secreted proteins. Most of these genes belonged to the GT (22) and GH families (20), and the rest were distributed among the AA (7) and CE (5) families. A growing body of evidence shows that glycosyltransferases play a vital role in hyphal growth, conidiation, stress responses, and human diseases by catalyzing glycosylation [28]. Glycoside hydrolases have recently been identified within the biosynthetic pathway of exopolysaccharides, which play an important role in biofilm formation by the opportunistic human pathogen, *Aspergillus fumigatus* [29].

A genome specificity analysis of *P. brasiliense* GGX 413 indicated that the specific genes were mainly related to the regulation of RNA biosynthesis and metabolic processes. According to relevant literature reports, a successful pathogen encounters numerous host-derived stresses when entering a mammalian host, and the modification of mRNA pools is used as a means of rapid adaptation to the host. The abundance of specific mRNAs changes rapidly, mediated by various RNA polymerase subunits, allowing the fungus to adapt to host-derived stresses [30].

## Conclusion

In summary, sequencing the genes of *P. brasiliense* GGX 413 provided a basis for predicting the structural characteristics and functions of its genome. We investigated the virulence factors, CAZymes, peptidases, secondary metabolites, and other gene products of *P. brasiliense* GGX 413 from a genetic perspective and screened its genome for pathogenic genes associated with human disease. We believe this approach will be useful for studying the underlying pathogenic mechanism of *P. brasiliense* GGX 413 and other fungi in humans in the future.

## Methods and materials

### Isolation and culture of strain

The left eyelid of a 71-year-old man was punctured by a tree branch after a fall; five days after the injury, the eyelid secretion was collected and used to inoculate Sabouraud agar plates (SDA CHROMagar) for the culture (at 25 °C) of any fungi present. The colony morphology of the pathogenic filamentous fungus was observed on days 4, 8, and 12 after inoculation. The mycelium was cultured for 4 days, observed and then transferred to potato dextrose broth medium (PDB Binhe Microorganism) for culture at 25 °C for 7 days with agitation at 200 rpm. The harvested mycelium was collected by centrifugation at 3000×*g*, and the supernatant was discarded.

### Strain identification

A growing filamentous fungal colony cultured in SDA medium for 5 days was sealed in a transport container and immediately transported to a commercial laboratory (Personalbio, Shanghai, China) for genomic DNA extraction, PCR amplification of internal transcribed spacer regions 1 and 2 (ITS 1 and 2), purification, and sequencing of the amplified product. The genomic DNA was extracted with the standard cetyltrimethyl ammonium bromide (CTAB) procedure. The forward and reverse primers for PCR amplification were ITS1 (TCCGTAGGTGAACCTGCGG) and ITS4 (TCCTCCGCTTATTGATATGC), and the reaction system (in a total volume of 50 µL) contained 1 µL of template DNA (20 ng/µL), 5 µL of 10 × buffer, 1 µL of *Taq* DNA polymerase (5 U/µL), 1 µL of dNTP (10 mM), 1.5 µL each of ITS1 and ITS4 primers (10 μM), and 39 µL of ddH_2_O. The PCR thermal cycling regimen was: initial denaturation at 95 °C for 5 min; 35 cycles of denaturation at 95 °C for 30 s, annealing at 58 °C for 30 s, and extension at 72 °C for 1 min; and a final extension reaction at 72 °C for 7 min. After the reaction, 3 µL of the PCR product was resolved with 1% agarose gel electrophoresis and purified strictly according to the instructions of the AxyPrep DNA Gel Recovery Kit (Corning). Sequencing was performed on the ABI 3730xl DNA Analyzer (Applied Biosystems). The BLASTN algorithm in the National Center for Biotechnology Information Nr database was used to construct a sequence alignment of various fungal ITS regions. A phylogenetic tree was constructed with MEGA-X, using the neighbor joining method.

### Sequencing and assembly

The filamentous fungus cultured in PDB medium for 7 days was collected and immediately transported to a commercial laboratory (Personalbio) for genomic sequencing and assembly. The genome was sequenced using a whole-genome shotgun strategy with next-generation sequencing on the Illumina NovaSeq platform and with third-generation sequencing on the Oxford Nanopore Technologies (ONT) platform. The genomic DNA was extracted with the CTAB method.

The total amount of DNA used for next-generation sequencing was determined according to the instructions of the Quant-iT™ PicoGreen™ dsDNA Assay Kit (Invitrogen), and the integrity of the DNA was checked with 1% agarose gel electrophoresis. A second-generation library was constructed based on the standard preparative procedure for an Illumina TruSeq DNA Nano LT library (Illumina TruSeq DNA Sample Preparation Guide). The quality of the library was assessed with the Agilent Bioanalyzer, according to the instructions of the Agilent High Sensitivity DNA Kit (Agilent Technologies Inc.), and the qualified library had only a single peak. The library was quantitatively analyzed with the Promega QuantiFluor® fluorescent quantification system, according to the standard protocol of the Quant-iT® PicoGreen® dsDNA Assay Kit, and the library concentration was acceptable, at > 2 nM. Libraries that met the quality requirements were sequenced. Paired-end sequencing was performed by adding sequencing primer, modified DNA polymerase, and dNTPs labeled with 4 fluorescence. Only a single dNTP was added in each cycle because the 3'-OH of the dNTP was blocked by an azide group. At the end of each cycle, the fluorescent group and azide group of the dNTP were chemically cleaved. A new dNTP was then added in the next cycle. The sequence information was analyzed from the fluorescent signals collected in each cycle. The quality control of the sequencing data was performed with FastQC (https://www.bioinformatics.babraham.ac.uk/projects/fastqc/). High-quality data were obtained by removing adapter contamination with Adapter Removal (version 2) [31], and all reads were quality corrected with SOAPec (version2.0) [32]. The sequencing data were assembled with Unicycler [33], and the assembled contigs were corrected with Pilon v1.18 [34]. The integrity and continuity of the assembled genome were assessed with BUSCO (version 3.0.2) [35].

The concentration and purity of the DNA used for third-generation sequencing were assessed with the Qubit™ dsDNA HS Assay Kit (Invitrogen) and a NanoDrop 2000 spectrophotometer (Thermo Fisher Scientific), and the integrity of the DNA was checked with 0.35% agarose gel electrophoresis. A third-generation sequencing library was constructed and sequenced with the standard protocol provided by ONT. The ONT sequencing analysis was based on the current signals. Under the traction of the motor protein, the DNA fragment bound to the nanopore protein embedded in the biofilm. After unwinding, the single-stranded DNA could pass through the nanopore channel protein at a certain rate under the action of the voltage difference on both sides of the biofilm, and different current signals were generated when different bases passed through the nanopore protein. Finally, a total of 528,651 reads with a GC content of 50.97% and an N50 fragment length of 26.1 kb were sequenced. The sequencing data were assembled with Unicycler [36].

### Genomic prediction and annotation

De novo gene prediction of the whole genome was performed with Augustus (version 3.03) [37], glimmerHMM (version 3.0.1) [38], and GeneMark-ES (version 4.35) [39]. Homologous genes were predicted based on the protein sequences in related species using Exonerate (version 2.2.0). The gene prediction results were integrated with EvidenceModeler (version r2012-06-25) [40]. Repeat sequences were identified by homologous annotation with RepeatMasker (version 4.0.5) [41] and annotated de novo with RepeatModeler (version 1.0.4) [42]. tRNA and rRNA genes were predicted with tRNAscan-SE (version 1.3.1) [43] and RNAmmer 1.2 [44], respectively, and other ncRNAs were mainly predicted by comparison with Rfam [45]. Pathogenicity-related genes were predicted with BLAST against the pathogen–host interactions (PHI) database and the database of fungal virulence factors (DFVF) with a cutoff *E*-value of 1e−6. The genes encoding carbohydrate-active enzymes (CAZymes) were predicted with a HMMER (version 3.1b2) scan against the profiles compiled with dbCAN release v10.0 [46] based on the CAZy database [47]. Secondary metabolite gene clusters were predicted with the antiSMASH database [48]. Annotations with EuKaryotic Orthologous Groups (KOG) were analyzed by aligning the genome against the eggNOG (evolutionary genealogy of genes, with Non-supervised Orthologous Groups) database, and using eggNOG-mapper with a cutoff *E*-value of 1e−6 [49]. Functional annotation with the Kyoto Encyclopedia of Genes and Genomes (KEGG) was performed with the KEGG automatic annotation server system (version 2.1) [50]. Functional annotation with Gene Ontology (GO) was performed with the InterPro and GOSlim (Generic) database [51]. The secretome was analyzed with the Fungal Secretome KnowledgeBase (FunSecKB) [52], and the peptidase proteins were checked against the MEROPS database (release 12.4) with a cutoff *E*-value of 1e−5 [53]. The gene family was analyzed with Orthofinder (version 2.2.7) with a BLASTP default value of -e = 0.001 [54].

### Phylogenetic tree analysis of single-copy gene families

A phylogenetic tree of single-copy gene families was generated using the alignment of 4995 single-copy orthologous gene families shared by 10 genomes (*P. minitans*, *P. sporulosa*, *Didymosphaeria enalia*, *Paraconiothyrium* sp., *P. brasiliense*, *Coniothyrium glycines*, *Paracamarosporium* sp., *Massarina eburnea*, *Byssothecium circinans*). The 4995 single-copy orthologous genes were aligned with Muscle. The phylogenetic tree matrix was constructed with the maximum likelihood method, with 100 bootstrap replications. The phylogenetic tree was visualized with the RAxML software [55].

## Supplementary Information

**Additional file 1: Table S1.** KEGG functional annotation of *P. brasiliense* GGX 413

**Additional file 2: Table S2.** The secretome of *P. brasiliense* GGX 413

## Data Availability

The metagenomic sequencing data have been deposited to the NCBI Sequence Read Archive (SRA) under the accession numbers SRR20089538 and SRR20067539. ITS sequence of *P. brasiliense* GGX 413 has been deposited to the NCBI GenBank under the accession number ON908491.

## Abbreviations

MAPK: Mitogen-activated protein kinase
p38: P38 MAP kinase
CYC: Cytochrome c
RAC1: Ras-related C3 botulinum toxin substrate 1
MAP2K1: Mitogen-activated protein kinase kinase 1
RHOA: Ras homolog gene family, member A
CDC42: Cell division control protein 42
PRKCA: Classical protein kinase C alpha type
GSK3B: Glycogen synthase kinase 3 beta
KRAS: GTPase KRas
PHI: Pathogen–host interactions
CAZymes: Carbohydrate-active enzymes
DFVF: Database of fungal virulence factors
KOG: EuKaryotic Orthologous Groups
KEGG: Kyoto Encyclopedia of Genes and Genomes
GO: Gene Ontology
